# Clinical efficacy of Enzyme Replacement Therapy in paediatric Hunter patients, an independent study of 3.5 years

**DOI:** 10.1186/s13023-014-0129-1

**Published:** 2014-09-18

**Authors:** Rosella Tomanin, Alessandra Zanetti, Francesca D’Avanzo, Angelica Rampazzo, Nicoletta Gasparotto, Rossella Parini, Antonia Pascarella, Daniela Concolino, Elena Procopio, Agata Fiumara, Andrea Borgo, Anna Chiara Frigo, Maurizio Scarpa

**Affiliations:** Department of Women’s and Children’s Health, University of Padova, Via Giustiniani, 3, 35128 Padova, Italy; Department of Paediatrics, Milano Bicocca University, San Gerardo Hospital, Monza, Italy; Department of Medicine Translational Sciences, Paediatrics Section, Federico II University, Napoli, Italy; Department of Paediatrics, Magna Graecia University of Catanzaro, Catanzaro, Italy; Metabolic and Muscular Unit, Clinic of Paediatric Neurology, Meyer Children’s Hospital, University of Florence, Firenze, Italy; Department of Paediatrics, Regional Referral Centre for Inborn Errors Metabolism, University Hospital “Policlinico-Vittorio Emanuele”, Catania, Italy; Department of Orthopaedics and Traumatology, University of Padua, Via Giustiniani, 2, 35128 Padova, Italy; Department of Cardiac, Thoracic and Vascular Sciences, University of Padova, Padova, Italy

**Keywords:** Enzyme Replacement Therapy, Hunter Syndrome, Lysosomal Storage Disorders, Paediatric populations, ERT efficacy, Long-term follow-up

## Abstract

**Background:**

Hunter Syndrome is an X-linked lysosomal storage disorder due to the deficit of iduronate 2-sulfatase, an enzyme catalysing the degradation of the glycosaminoglycans (GAG) dermatan- and heparan-sulfate. Treatment of the disease is mainly performed by Enzyme Replacement Therapy (ERT) with idursulfase, in use since 2006. Clinical efficacy of ERT has been monitored mainly by the Hunter Outcome Survey (HOS) while very few independent studies have been so far conducted. The present study is a 3.5-years independent follow-up of 27 Hunter patients, starting ERT between 1.6 and 27 years of age, with the primary aim to evaluate efficacy of the therapy started at an early age (<12 years).

**Methods:**

In this study, we evaluated: urinary GAG content, hepato/splenomegaly, heart valvulopathies, otorinolaryngological symptoms, joint range of motion, growth, distance covered in the 6-minute walk test, neurological involvement. For data analysis, the 27 patients were divided into three groups according to the age at start of ERT: ≤5 years, >5 and ≤ 12 years and > 12 years. Patients were analysed both as 3 separate groups and also as one group; in addition, the 20 patients who started ERT up to 12 years of age were analysed as one group. Finally, patients presenting a “severe” phenotype were compared with “attenuated” ones.

**Results:**

Data analysis revealed a statistically significant reduction of the urinary GAG in patients ≤5 years and ≤ 12 years and of the hepatomegaly in the group aged >5 and ≤ 12 years. Although other clinical signs improved in some of the patients monitored, statistical analysis of their variation did not reveal any significant changes following enzyme administration. The evaluation of ERT efficacy in relation to the severity of the disease evidenced slightly higher improvements as for hepatomegaly, splenomegaly, otological disorders and adenotonsillar hypertrophy in severe vs attenuated patients.

**Conclusions:**

Although the present protocol of idursulfase administration may result efficacious in delaying the MPS II somatic disease progression at some extent, in this study we observed that several signs and symptoms did not improve during the therapy. Therefore, a strict monitoring of the efficacy obtained in the patients under ERT is becoming mandatory for clinical, ethical and economic reasons.

**Electronic supplementary material:**

The online version of this article (doi:10.1186/s13023-014-0129-1) contains supplementary material, which is available to authorized users.

## Background

Hunter Syndrome (Mucopolysaccharidosis type II, MPS II) is a rare, X-linked, inherited, lysosomal storage disorder with an estimated incidence of 1.3 in 100.000 male newborns [1]. It is due to the deficit of activity of the lysosomal enzyme iduronate 2-sulfatase (IDS), normally degrading heparan- and dermatan-sulfate within lysosomes. Insufficient or, commonly, totally absent levels of IDS activity lead to progressive accumulation of these GAG species in nearly all cell types, tissues, and organs of the body, including respiratory tract, heart, liver, spleen, bones, joints, oropharynx, head, neck, leptomeninges and central nervous system (CNS) [2].

Hunter Syndrome is always a progressive, chronic and life-threatening condition. Clinical manifestations vary considerably from patient to patient. However, two major phenotypes are formally recognized, a severe and an attenuated form, mainly differing for the lack of the CNS involvement in the latter, also characterized by a slower progression of the disease. Onset of signs and symptoms typically occurs between 18 months and 4 years of age in the severe phenotype and about 2 years later in the attenuated form [2-4]. The most common peripheral clinical signs and symptoms include coarse facial features, hearing loss, restrictive lung disease, hepato/splenomegaly, heart valvulopathy, decreased joint range of motion, skeletal deformities and short stature. In addition, oropharyngeal and respiratory deposition of GAG leads to severe airways obstruction, further contributing to impaired pulmonary function and sleep apnoea. About two-thirds of the patients present involvement of the CNS, leading to progressive severe mental retardation, often in association with communicating hydrocephalus and increased intracranial pressure, which may also affect the attenuated forms [5]. Due to a combination of the bone disease, decreased respiratory capacity and sleep apnoea, together with impaired cardiac function, patients with Hunter Syndrome suffer from chronic, severely impaired endurance. As disease progresses their ability to walk may be partially lost or for many patients totally lost. In the later stages of the disease, the continuous accumulation of GAG leads to progressive organ failure and significantly shortened lifespan. Death usually occurs in the second or third decade of life or even later for the attenuated forms, most often from respiratory and/or cardiac failure [2,3].

Haematopoietic transplant, applied mainly in the past, has shown poor results [6,7]. The full cloning of the IDS sequence has allowed the production of the recombinant form of the enzyme and its administration with an Enzyme Replacement Therapy (ERT) protocol. ERT was fully licensed for MPS II by the USA FDA in 2006 and in the same year Italy was the first country in Europe to provide the drug to the patients. Since the first clinical trial, performed in the USA in 2005, the Hunter Outcome Survey (HOS), supported by Shire HGT, Inc., has collected data on Hunter patients under ERT with the objective to define the natural history of the disease and to monitor safety/efficacy of the treatment. However, several aspects still need to be fully evaluated. One of the principal issues to address is to understand whether the treatment started at an early age might result in a better prognosis. As stated by Muenzer [8] the rationale of an early initiation of ERT is supported by several observations: demonstrated lysosomal GAG storage in prenatal age, improvements in precociously treated animal models, case reports of siblings treated at different ages, etc. The Phase I/II and II/III clinical trials enrolled only patients older than 5 years and able to comply with the repeated pulmonary function and endurance testing [9,10]. Recently, two studies [11,12] on children younger than 6 years were published with data from HOS. In the first study [11] 6 children who started ERT from 2.8 to 4.7 years of age, treated for a mean period of 9 months, evidenced some beneficial effects from ERT. Reduction in urinary GAG levels, small reductions in liver and spleen volumes, improvement or stabilization of joint mobility were the main observed effects. The second study [12] included 124 patients younger than 6 years at start of therapy and followed for a mean period of 23 months; however, this study only provided data related to urinary GAG levels, hepatomegaly and antibodies response. In addition, a multicentre open-label study [13] conducted by Shire in children aged 1.4 to 7.5 years, evidenced a safety profile similar to that observed in patients ≥5 years and reduction of liver and spleen size and of urinary GAG after 1 year of treatment. Finally, a study on patients under 1 years of age was recently performed by Lampe [14] and colleagues evidencing, after a treatment longer than 6 weeks, an improvement or a stabilization of some somatic symptoms. Instead, very few independent investigations have been so far conducted; among these, a recent British study funded by the Health Technology Assessment (HTA) program [15]. The study recruited 36 paediatric patients aged < 16 years (range 2.3-15.6) and 3 adults, and evaluated Forced Vital Capacity (FVC), mobility, 6-Minute Walking Test (6MWT), height, weight, hearing, heart valve disease, carpal tunnel syndrome and spleen and liver size, after 12 and 24 months of therapy. Data analysis revealed only a statistically significant association between duration of ERT and height measurements.

The present study, supported by AIFA (Italian Medicines Agency), is the first independent data collection carried on for a quite long time (about 3.5 years) after the start of idursulfase ERT. It has the aim to evaluate the efficacy of the recombinant enzyme administration, by analysing several clinical and laboratory parameters in a paediatric population aged between 1.6 and 12 years at start of ERT, together with an additional Hunter group starting ERT between 12 and 27 years of age.

## Methods

### Study inclusion criteria and clinical data collection

Twenty-seven patients were progressively enrolled in the study from six different Clinical Units according to the following inclusion criteria: diagnosis of MPS II confirmed by low/absent IDS activity in leukocytes or fibroblasts, a normal activity of other sulphatases, thus excluding multiple sulphatase deficiency, high levels of heparan- and dermatan-sulphate in urine.

Informed consent to the participation in the study was obtained from the patients or their legal tutors, who maintained at any time full possibility to retire their consent with no interruption of the appropriate medical care and ERT administration.

The study was previously submitted to the Institutional Review Board/Ethical Committee of each single Clinical Unit which gave ethical approval to the enrollment of the patients in the study:

- Azienda Ospedaliera di Padova - Comitato Etico (approval date: 15/09/2008)
- Azienda Ospedaliera “Pugliese-Ciaccio” Catanzaro - Comitato Etico (approval date: 06/06/2009)
- Università degli Studi di Napoli Federico II - Comitato Etico per le Attività Biomediche (approval date: 30/09/2009)
- Azienda Ospedaliero-Universitaria “Policlinico Vittorio Emanuele” Catania - Comitato Etico (approval date: 27/06/2011)
- Azienda Ospedaliero-Universitaria Meyer Firenze - Comitato Etico per la Sperimentazione dei Medicinali (approval date: 16/09/2011)
- Azienda Ospedaliera San Gerardo Monza - Comitato Etico (approval date: 15/12/2011).

Idursulfase (Elaprase^®^, Shire Human Genetics Therapies Inc., USA) was weekly infused in 3 hours time at the dosage of 0.5 mg/kg of body weight, appropriately diluted in 0.9% sodium chloride according to producer’s indication. At each infusion, vital signs were assessed in four different moments: immediately prior to infusion, after an hour of infusion, at the end of the infusion and one hour later.

In addition to ERT, each patient continued to receive the appropriate medications, previously established, to manage chronic or acute symptoms related to the disease.

The criteria for selection of the clinical outcome measures or variables for the assessment of ERT efficacy were guided by the principle that only those indicators reflecting the extent of disease progression could be useful to determine the efficacy of the treatment.

As for laboratory parameters, the evaluation of urinary GAG content was performed.

For the clinical assessments, evaluation of the presence/absence of specific signs and symptoms were performed before the beginning of ERT, during ERT at determined time-intervals and at the end of the follow-up. The following signs and symptoms were evaluated: hepatomegaly and splenomegaly (assessed through abdomen ultrasound), heart valvulopathies (through echocardiography), otological disorders, adenotonsillar hypertrophy and the related sleep disturbances (through audiometric and otolaryngological evaluations), joint range of motion (through orthopaedic and physiatric evaluations). The neuroradiological alterations typical of the disorder [white matter abnormalities (WMAs), cerebral atrophy, ventricular dilation, perivascular alteration] were evidenced through neuroimaging. Different cognitive tests were administered according to the patient’s age and disease severity, 6MWT was administered only in collaborating patients. In addition, height and weight measures were collected.

In addition to previously listed variables, we also registered other data including routine laboratory haematochemical parameters, raising of anti-IDS antibodies, pubertal development, measurement of head circumference, eye evaluation. All data were collected through a Case Report Form (CRF) previously approved by our Institutional Review Board.

A follow-up was conducted for an average of 3.3 ± 1.5 years (median = 3.3 years). Pre-ERT evaluations were considered those taken as proximate as possible to the start of ERT, while as last we chose evaluations closest to 3.5 years from the start of the therapy, when available. While the total number of enrolled patients was 27, not all of them were included in each evaluation; consequently, the total number of patients analysed ranges from 9 to 25, according to the variable considered.

### Criteria for data analysis

Due to the lack of a comprehensive reconstruction of the natural history of the disease, and to the unavailability of a control ERT naive group (Hunter patients not undergoing ERT), we considered the treatment effective when it determined “an improvement in or a prevention of progression of disease activity as indicated by a stabilization in clinical condition together with an improvement in the abnormalities present at baseline” as suggested by Vellodi et al. 2010 [16].

For data analysis, two different stratifications of the patients were performed: by age at start of ERT and by disease severity.

As for the age at start of ERT, the following groups were considered:

1. group A (≤5 years, n = 13), group B (>5 and ≤ 12 years, n = 7), group C (>12 years, n = 7) separately analysed;
2. patients ≤12 years of age taken together (group A + B; n =20);
3. all patients analysed as a single group (group A + B + C; n = 27).

As for disease severity, patients were divided according to their “severe” or “attenuated” phenotype as previously described [4].

All analyses were performed by comparing pre-ERT with post- ERT data.

### Statistical data analysis

Since urinary GAG were measured by applying different methodologies and measurement units by the different Operative Units, the percentage ratio of each considered time-point from pre-ERT baseline value, taken as 100%, was calculated. The analysis was conducted with the Wilcoxon signed rank test at 1, 2 and 3 years post-ERT and at the last available evaluation.

Data related to hepatomegaly, splenomegaly, otological disorders, adenotonsillar hypertrophy, sleep disturbances, CNS abnormalities by brain imaging, cognitive function involvement and seizure were reported as dichotomous variables representing the presence or the absence of a given pathological phenotype before treatment and at the end of the study. The variation pre-post treatment was analysed with the McNemar test. Therapeutic efficacy was evaluated as proportion of positive outcomes, intended as the number of patients for which the pathological phenotype was improved (feature present before the start of therapy and disappeared after treatment) plus those patients for which the phenotype did not worsen (feature absent both before and after therapy). The confidence intervals for this proportion was calculated by the method of Clopper and Pearson [17].

As valvulopathies we considered valve regurgitation of each cardiac valve, and reported its severity according to the scale suggested by Zoghbi [18].

For joint mobility, therapeutic efficacy was also evaluated by assigning a judgment of stabilization (s), worsening (w) or improvement (i) of the clinical status of the patient with respect to the pre-ERT situation, taking into account the evaluations of joint range of motion and all inherent available data in the orthopaedic history of each patient. The proportions of each of these classes (s, w, i) are reported with confidence intervals calculated as above.

Growth was evaluated by z-score (Standard Deviation Score, SDS) calculated for each height and weight measurements using the 2000 Centre for Disease Control LMS parameters (http://www.cdc.gov/growthcharts/percentile_data_files.htm) according to the formula reported by Cole and Green [19]. A first order autoregressive model, AR(1), was used to analyse the growth data.

In the 6MWT performance, the distance covered by the patients under study was compared with normal values for children aged 4–11 taken from Lammers, 2008 [20].

All statistical tests were conducted at the level of significance of 5%. Due to the exploratory hypothesis-generating rather than hypothesis-testing nature of the study, no correction to the p-values was applied for the multiplicity of the analyses taken.

## Results

### Main features of the population examined

Twenty-seven patients were enrolled. All patients completed the study, except one because of his exitus caused by cardiopulmonary arrest at 25 years of age.

Basic information related to the 27 patients enrolled is reported in Table 1. Most patients presented with a severe phenotype (17 out of 27), 10 showed an attenuated phenotype. The genetic characterization revealed 14 different missense, 3 nonsense and 2 splicing mutations, 3 small and 1 large deletions, and 1 IDS-IDS2 recombination. In only one patient the mutation was not identified. In the cohort of patients analysed, two couples of siblings were present (A3 and A11; A7 and A10). Apart from siblings, all patients, except 2, showed different mutations. The only 2 non-sibling patients carrying the same genomic variant (B5, C7) presented an attenuated and a severe phenotype, respectively.

**Table 1:** **Characteristics of the patients enrolled in the study**

**Patient ID**	**Phenotype**	**Type of mutation**	**Age at diagnosis**	**Age at start of ERT**
**Group A**				
A1	Severe	Intragenic deletion	0.9	1.6
A2	Severe	Missense	1.0	1.6
A3	Severe	Missense	2.2	2.3
A4	Attenuated	Small deletion	1.8	2.4
A5	Attenuated	Splicing mutation	2.4	2.6
A6	Severe	Small deletion	2.8	2.9
A7	Attenuated	Two *in cis* missense	1.3	3.3
A8	Severe	Missense	2.8	3.4
A9	Severe	Splicing mutation	3.2	3.4
A10	Attenuated	Two *in cis* missense	n.a.	3.7
A11	Severe	Missense	3.5	3.7
A12	Severe	Missense	4.4	4.7
A13	Severe	Missense	4.7	5.0
**Group B**				
B1	Severe	Small deletion	2.5	5.3
B2	Attenuated	Nonsense	n.a.	6.9
B3	Severe	Missense	2.2	7.7
B4	Severe	Recombination	3.1	8.0
B5	Attenuated	Nonsense	4.5	9.0
B6	Severe	Missense	7.1	9.2
B7	Severe	Missense	7.8	11.4
**Group C**				
C1	Severe	Missense	4.3	12.7
C2	Severe	Nonsense	4.0	15.8
C3	Attenuated	Missense	3.8	16.3
C4	Attenuated	n.i.	6.8	16.7
C5	Attenuated	Missense	15.5	18.7
C6	Attenuated	Missense	15.2	18.8
C7	Severe	Nonsense	4.1	27.0

Age at start of ERT: group A ≤5 years, group B >5 and ≤ 12 years, group C >12 years (n.i. = not identified).

The age at diagnosis was available for 25 patients and ranged from 0.9 to 15.5 years (median 3.5). The age at start of ERT was between 1.6 and 27 years with a median age of 5.3.

### Urinary glycosaminoglycan levels

**Figure 1:**
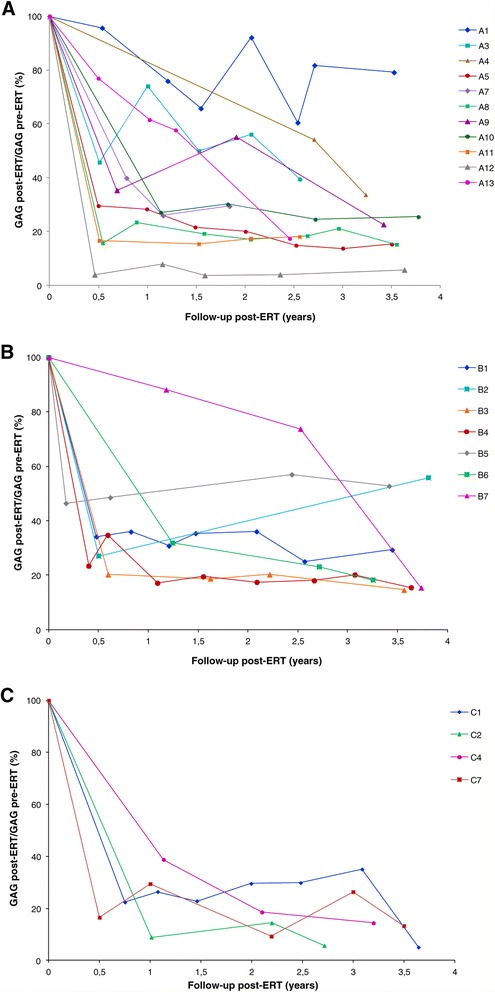
**Urinary GAG dosage.** Urinary GAG values are reported for each subject as percentage with respect to the pre-ERT level. **A**: patients aged ≤5 years, **B**: patients aged >5 and ≤ 12 years, **C**: patients aged >12 years.

Figure 1 reports urinary GAG levels measured at several time-points in the course of the follow-up for the 3 groups of patients. Data were collected for a median time of 3.2 (0.4-3.8), 3.6 (3.3-3.8) and 3.4 (2.7-3.6) years for groups A, B and C respectively, for 25 out of 27 patients.

Table 2 reports the results of the statistical data analysis performed on GAG percentage variation calculated with respect to pre-ERT GAG values.

**Table 2:** **Statistical analysis of GAG percentage variation from pre-ERT values**

**Age group**	**Time point from start of ERT**	**n**	**Median (minimum-maximum)**	**p-value**
**A**	1 yr ± 4 m	12	37.6 (7.8-76.0)	0.0005*
	2 yrs ± 4 m	9	29.4 (3.9-92.1)	0.0039*
	3 yrs ± 4 m	7	24.5 (13.7-81.8)	0.0156*
	Last	13	25.4 (5.7-79.2)	0.0002*
**B**	1 yr ± 4 m	6	33.9 (17.1-88.1)	0.0313*
	2 yrs ± 4 m	4	19.5 (17.4-36.0)	0.1250
	3 yrs ± 4 m	4	19.2 (18.0-23.1)	0.1250
	Last	7	18.2 (14.6-55.8)	0.0156*
**C**	1 yr ± 4 m	5	26.4 (8.9-38.8)	0.0625
	2 yrs ± 4 m	4	16.5 (9.3-29.6)	0.1250
	3 yrs ± 4 m	5	26.4 (5.7-35.1)	0.0625
	Last	5	13.3 (5.1-28.2)	0.0625
**A + B**	1 yr ± 4 m	18	35.6 (7.8-88.1)	< .0001*
	2 yrs ± 4 m	13	20.2 (3.9-92.1)	0.0002*
	3 yrs ± 4 m	11	21.0 (13.7-81.8)	0.0010*
	Last	20	24.0 (5.7-79.2)	< .0001*
**A + B + C**	1 yr ± 4 m	23	31.8 (7.8-88.1)	< .0001*
	2 yrs ± 4 m	17	20.0 (3.9-92.1)	< .0001*
	3 yrs ± 4 m	16	22.0 (5.7-81.8)	< .0001*
	Last	25	18.2 (5.1-79.2)	< .0001*

Urinary GAG values are expressed at each time-point, from start of ERT as percentage with respect to the pre-ERT baseline level, taken as 100%. The number of available samples considered in the calculation (n), the median value and minimum and maximum values, together with the p-value of the statistical test applied (Wilcoxon signed rank test), are reported. Statistically significant values (p < 0.05) are marked with an asterisk.

The variations in GAG levels resulted significant at each time-point when considering all patients together and the group of patients up to 12 years of age at start of ERT. The separate evaluation of each age group also gave statistically significant results at all time-points only for group A; evaluations of group B was significant after 1 year of treatment and at the last evaluation, while for group C data were not statistically significant at all the time-points considered.

### Hepatomegaly and splenomegaly

The absence-presence of hepatomegaly and splenomegaly, evidenced by abdominal ultrasound, before the beginning of the therapy and at the end of the follow-up was registered. The median follow-up duration was 2.9 (1.6-3.7) years for group A, 3.3 (1.4-3.6) years for group B and 3.2 (0.8-3.6) years for group C. Data are separately reported for liver and spleen in Tables 3 and 4 respectively, together with the statistical analysis. The McNemar test applied highlighted a significant amelioration of hepatomegaly only for the patients of group B with 6 out of 7 patients presenting no liver enlargement at the end of the study (Table 3). No significant improvements were evidenced for splenomegaly in all groups analysed (Table 4).

**Table 3:** **Contingency table and statistical analysis of hepatomegaly data**

**Group**	**Pre-post**				**McNemar test p-value**	**Positive Outcomes**	
	**Y-Y**	**Y-N**	**N-Y**	**N-N**		**Proportion**	**CI (95%)**
**A**	3	3	4	1	1.0	0.36	(0.109, 0.692)
**B**	1	6	0	0	0.03*	0.86	(0.421, 0.996)
**C**	3	2	0	2	0.50	0.57	(0.184, 0.901)
**A + B**	4	9	4	1	0.27	0.56	(0.308, 0.785)
**A + B + C**	7	11	4	3	0.12	0.56	(0.349, 0.756)

The category “Positive Outcomes” includes both YES-NO (Y-N: improvement) and NO-NO (N-N: stabilization) cases. Confidence intervals, calculated by the Clopper and Pearson method and the p-value of McNemar test are reported. Statistically significant values (p < 0.05) are marked with an asterisk. Group A: n = 11, group B: n = 7, group C: n = 7.

**Table 4:** **Contingency table and statistical analysis of splenomegaly data**

**Group**	**Pre-post**				**McNemar test p-value**	**Positive outcomes**	
	**Y-Y**	**Y-N**	**N-Y**	**N-N**		**Proportion**	**CI (95%)**
**A**	0	1	3	5	0.63	0.67	(0.299, 0.925)
**B**	2	2	0	2	0.50	0.67	(0.223, 0.957)
**C**	2	3	0	2	0.25	0.71	(0.290, 0.963)
**A + B**	2	3	3	7	1.00	0.67	(0.384, 0.882)
**A + B + C**	4	6	3	9	0.51	0.68	(0.451, 0.861)

The category “Positive Outcomes” includes both YES-NO (Y-N: improvement) and NO-NO (N-N: stabilization) cases. Confidence intervals, calculated by the Clopper and Pearson method and the p-value of McNemar test are reported. Group A: n = 9, group B: n = 6, group C: n = 7.

Analysis of all patients taken together (A + B + C) and of all patients under 12 years of age (A + B) did not reveal any significant improvements for both organs analysed.

### Cardiac valve disease

**Figure 2:**
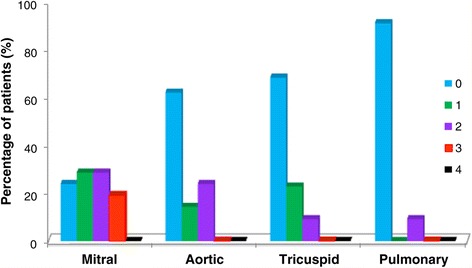
**Pre-ERT cardiac valve regurgitation.** Percentage of patients presenting valve regurgitation and related degree of severity according to Zoghbi et al. [18], before the beginning of ERT (n =21 for mitral and aortic valves, n = 22 for tricuspid and pulmonary valves).

Figure 2 shows the prevalence and the severity of valve regurgitation in the 27 patients enrolled in the study, before the beginning of the therapy, evidenced through echocardiography. The mitral valve was the most involved with 76% of the subjects affected, with a severity score from 1 to 3; 38% and 32% of the patients presented with regurgitation of the aortic and of the tricuspid valves respectively (score 1÷2) while only in 9% of them an involvement of the pulmonary valve was observed.

**Figure 3:**
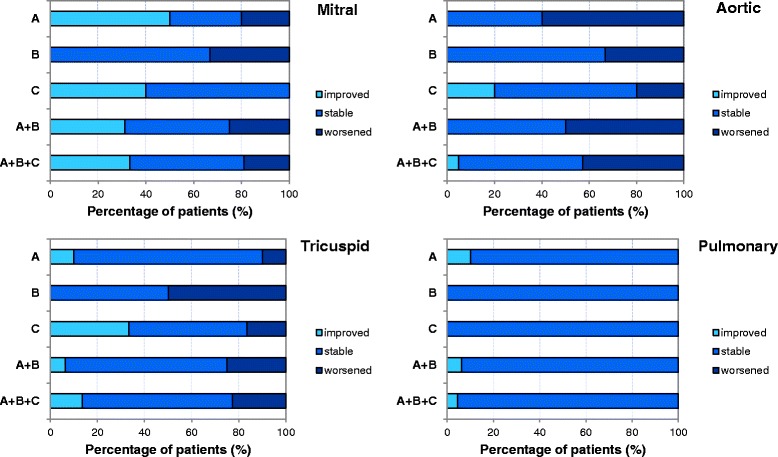
**Post-ERT cardiac valve regurgitation.** Percentage of patients of group A, B, C, A + B and A + B + C presenting an improvement, a stabilization or a worsening of regurgitation of the mitral, aortic, tricuspid and pulmonary cardiac valves. Group A: n = 10, group B: n = 6, group C: n = 6 for tricuspid and pulmonary valves, n = 5 for mitral and aortic valves.

Figure 3 reports for each valve the percentage of patients for group A, B, C, A + B and A + B + C presenting an improvement, a stabilization or a worsening of regurgitation after a median follow-up of 2.9 (1.6-3.8) years for group A, 3.4 (0.6-3.7) years for group B and 3.0 (1.0-3.7) years for group C. The highest frequency of positive outcomes was observed for the mitral valve with most patients of all groups presenting an improvement or a stabilization. A stabilization of the aortic valve regurgitation was observed for most groups except for group A, for which a worsening of the condition was registered. Also for the tricuspid valve a stabilization of the condition for group A, A + B and A + B + C, or a balance between worsening and stabilization (group B and C) were registered, while the high percentage of stabilization observed in the pulmonary valve is ascribable to the absence of involvement evidenced both before and after therapy in all groups of patients.

### ENT manifestations

Among all Ear, Nose and Throat (ENT) manifestations, otological disorders, adenotonsillar hypertrophy and sleep disorders of respiratory origin were taken into consideration. Such variables were evaluated at pre-ERT time, when available, and at the end of the study with a median follow-up time of 3.0 (0.1-3.8) years for group A, 3.8 (1–4.7) years for group B and 4.1 (3.2-5.3) years for group C.

Data are reported in the contingency tables, together with the statistical analysis, in Additional file 1. As for otological disorders, none of the 3 groups separately analysed showed a statistically significant improvement at the end of the follow-up. About 50% of the entire population (A + B + C) and 40% of the patients aged ≤12 years (A + B) showed an improvement of the otological disease, or at least did not register a worsening of the ear condition in the course of the treatment; however, this did not result in any statistical significance.

Evaluation of adenotonsillar hypertrophy was performed only for patients that did not undergo adenotonsillectomy. No statistically significant amelioration was observed for the 3 groups separately analysed. In addition, the analysis of the whole population examined and of the patients under 12 years of age did not show any statistical significance.

As for sleep disturbances of respiratory origin, the separate analysis of the 3 groups did not evidence any statistically significant amelioration due to the treatment, as well as the analysis of the combined groups A + B + C and A + B.

### Growth

**Figure 4:**
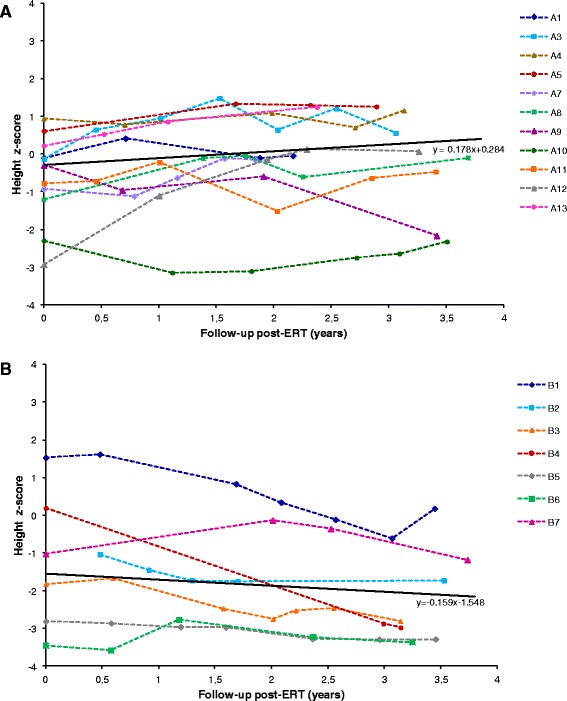
**Height z-scores.** Regression plot for height z-scores before the start of ERT (time-point = 0) and after the start of ERT (time-points t >0) for patient groups aged ≤5 years **(A)** and >5 and ≤ 12 years **(B)**.

To evaluate growth of children affected by MPS II and treated with idursulfase, we collected height measurements during the follow-up and calculated the z-scores for each time-point for all patients of groups A and B (Figure 4). Due to the older age, no height curves could be analysed for the patients of group C. The regression plot for group A is represented by a line with a slight positive slope indicating that all patients tend to overcome the mean value over the time, although oscillating around the average (z-score = 0). On the opposite, group B data are interpolated by a negative slope line located below the average for all time-points considered (z-score = −1.5÷−2), suggesting a decreasing growth rate for this age group.

Data on weight for groups A and B are provided as Additional file 2. In group A the regression line of weight z-scores is positioned slightly above the average (z-score = 0.53÷0.63) and is quite stable over the time. On the opposite, in group B the slope line is negative as Z-score values decrease during the follow-up from about 0.9 to 0.2.

### Orthopaedic evaluation

**Figure 5:**
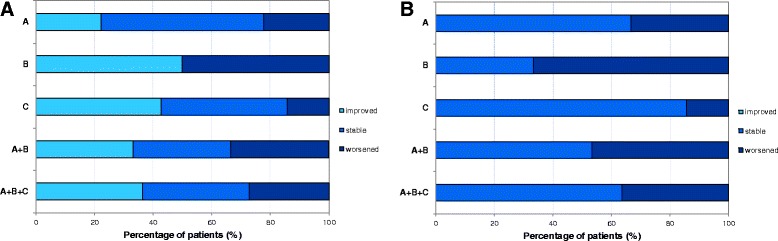
**Evaluation of joint stiffness.** Percentage of patients of group A, B, C, A + B and A + B + C presenting an improvement, a stabilization and a worsening of joint stiffness for upper **(A)** and lower limbs **(B)**.

The evaluation of joint range of motion (JROM) of upper limbs (shoulder and elbow) and lower limbs (knee and ankle) is provided in Figure 5A and B respectively, and it is expressed as stabilization, worsening or improvement of JROM with respect to the pre-ERT clinical picture. One-third of the patients aged <12 years showed an amelioration of upper limbs movements while no improvements were observed in lower limbs. In group C, 43% of patients evidenced an increased mobility of upper limbs, while no patients showed improvements of lower limbs. Hence, where a reduction of joint limitation was registered this involved almost exclusively the upper limbs, in particular the shoulders (with improved range of abduction), as clearly evidenced in Figure 5A, although no statistically significant amelioration was observed for any of the groups analysed.

Statistical analysis of the entire population examined (A + B + C) and of each single age group taken separately (A, B, C) also did not highlight significant improvement of JROM due to the treatment.

### Distance covered in the 6-Minute Walk Test (6MWT)

**Figure 6:**
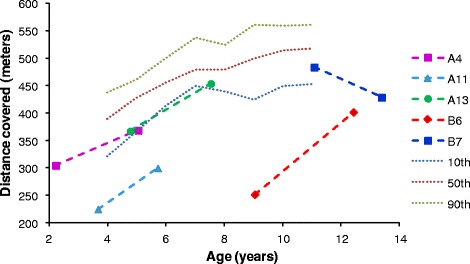
**Distance covered in the 6MWT.** Distance covered before and after ERT. Reference values for healthy children aged 4–11 years, represented as dotted lines, were taken from Lammers et al. 2008 [20].

This endurance test was administered rarely to the patients during the follow-up. The principal reason dealt with the difficulty to obtain collaboration by the patients, in particular in the youngest group. In addition, during the follow-up most patients registered a worsening of the psyco-motor delay with the consequent inability to walk. Therefore, data are only available for 6 patients (Figure 6): 3 from group A, 2 from group B and 1 from group C (this last one not is reported in the figure due to unavailability of data of an age-paired healthy population). In group A, all 3 patients showed a mean improvement of about 20% on the distance covered; since this improvement might be partly due to the increasing age and related ability to walk, data obtained were compared with age-related percentile of an healthy population. In group B, 1 patient improved (+37%) and the second one reduced (−13%) the stretch walked. The single patient examined from group C improved his performance of about 20%.

Overall, although a positive trend could be observed, the paucity of data did not allow to draw any conclusions about the efficacy of the therapy on this performance.

### Neurological evaluation

Neurological analysis, including brain imaging, cognitive tests and evaluation of seizures, is reported in Additional file 3.

All patients of all groups presented neurological alterations typical of the disorder, detected by neuroimaging; in particular, 2 patients (1 for each group A and B) manifested hydrocephalus requiring ventriculoperitoneal shunting; due to the appearance of a marked stenosis of the cervical medulla, a second patient from group A underwent decompression surgery. In all 3 cases surgery was performed after the start of ERT.

In group A, cognitive deterioration could be observed in 7 patients out of 11; 4 of them showed, at baseline, borderline or mild cognitive delay, apparently stabilized, thus we considered these cases as “attenuate” phenotypes. In group B, 4 patients presented a cognitive decline while 2 patients showing a stable profile of mild mental delay were assigned to the attenuated phenotype. In group C, 3 patients presented a preserved cognitive function which allowed a regular schooling, including university studies for 2 of them.

In two patients of group A, seizures appeared during ERT follow-up. In groups B and C, 5 more patients showed seizures, already present in the pre-ERT phase for 4 of them, while for 1 subject pre-ERT data were not available. All subjects presenting seizures also showed cognitive impairment.

No statistically significant amelioration was obtained for any of the parameters observed; also the analysis of A + B + C or A + B groups did not produce any statistically significant results.

### Other evaluations

Biochemical and haematochemical laboratory parameters regularly examined did not generally show notable alterations.

The measurement of the FVC, scheduled for patients aged 6–12 years, was rarely administered due to poor collaboration of the patients because of their psychomotor delay and cognitive impairment.

For the remaining evaluations reported in the Materials and Methods section, no thorough analysis was performed, given the incomplete set of data available.

### ERT efficacy evaluation in severe versus attenuated phenotypes

In Additional file 4 we report the analysis of the data obtained by subdividing patients into severe (n = 17) and attenuated (n = 10) phenotype. The reduction of urinary GAG levels resulted significant at each time-point for severe patients, after 1 and 3 years of treatment and at the last evaluation for the attenuated ones. Moreover, the proportion of positive outcomes obtained by ERT was found to be higher in severe vs attenuated patients for several parameters. These included hepatomegaly (60% of severe vs 50% of attenuated), splenomegaly (76.9% of severe vs 55.6% of attenuated), otological disorders (58.3% of severe vs 33.3% of attenuated), adenotonsillary hypertrophy (75% of severe vs 20% of attenuated) and all valve regurgitations except the mitral one (57% severe vs 86% attenuated). No differences in the percentage of improved patients were observed for joint stiffness between the 2 groups, since the great majority of the patients did not obtain any ameliorations from ERT as well as for the CNS abnormalities detected by brain imaging. Finally, attenuated patients presented no cognitive function involvement, as expected, no ENT-related sleep disturbances and no seizures.

## Discussion

Until recently, treatment of Hunter Syndrome was only palliative and focused on clinical symptoms. Nowadays treatment is mainly performed by an Enzyme Replacement Therapy (ERT) protocol, employing a recombinant form of idursulfase. Safety and efficacy of ERT have been primarily evaluated by two randomized trials [9,10] and subsequently by an open-label extension of the trial [21]. Since 2005 the HOS collects data on Hunter patients with the intent to delineate the natural history of the disease and to monitor safety and effectiveness of ERT. However, several aspects still have to be fully considered. One of the main issues is to understand whether early treatment might importantly slow down the disease progression, given that criteria for enrolment in clinical trials did not imply any restrictions related to the age of the patients. In addition, when the drug was released the majority of Hunter patients went on treatment at any age; therefore an extensive evaluation of the efficacy on paediatric patients remains important to perform. To this aim, we here analysed, as an independent evaluation, the efficacy of ERT, performed with Elaprase^®^, administered weekly at a dosage of 0.5 mg/kg in a paediatric population starting treatment earlier than 12 years of age. In parallel, a small population of subjects, starting ERT later than 12 years of age, was enrolled.

In the last years two HOS reports on children younger than 6 years were published [11,12]; however these studies presented several limitations mainly related to the small number of patients evaluated [11] and/or the limited number of variables analysed [12].

Up to date, few independent reports were published, among which a recent British study funded by the HTA program [15], analysing 36 patients below 16 years of age and 3 adults which evidenced only height improvements, significantly associated to the duration of ERT. The authors, however, stated that some of the variables assayed were “hampered by a paucity of data related to both the small number of affected patients recruited and lack of recording of data on key outcomes for a substantial proportion of the patients” [15].

The present study enrolled 27 patients, clustered in 3 groups according to the age at start of ERT (≤5 years (group A), >5 and ≤12 years (group B and >12 years (group C)) and classified as “severe” (17 cases) or “attenuated” (10 cases), on the basis of the clinical and neurological evaluations. The percentage of severe patients was therefore 63%, a proportion similar to that commonly described [22]. Our population included 2 couples of siblings. The 2 subjects of each couple, though presenting the same mutation and a likely similar genomic background, showed a different level of involvement for some of the organ systems analysed. Interestingly, 2 non-sibling patients showing the same nonsense mutation presented an attenuated and a severe phenotype, respectively. This confirms the poor genotype-phenotype correlation widely recognized for the disorder [23], which usually complicates or hampers prognosis on phenotype severity and/or disease progression.

Our analysis on the efficacy of this ERT protocol included several clinical parameters as well as laboratory measurements mostly related to important peculiar clinical signs of MPS II; thus, the chosen parameters possibly reflected the extent of disease progression.

In both groups of patients examined, aged ≤ 12 years, we observed a significant urinary GAG reduction, while such significant decrease was not measured in group C. However, this result may be importantly affected by the small number of patients examined in group C. Urinary GAG decrease is widely used as indicator of therapeutic efficacy in MPS II patients. However, how a decrease of urinary GAG level might be related to the efficacy of the drug on tissue and organ systems is still to be defined [24]. Based on our observations, not always a decrease of urinary GAG corresponds to an improvement of other signs, as hepato and/or splenomegaly or to a general amelioration of the clinical phenotype. Urinary GAG measurement is a simple, quite inexpensive and non-invasive procedure useful as diagnostic tool; its value for treatment monitoring should be re-evaluated or, at least, GAG results should be supported by the parallel evaluation of a second parameter.

Analyses of hepatomegaly and splenomegaly were performed separately in our evaluation. Distribution of the patients in groups of increasing age allowed to observe that hepatomegaly can be detected in the course of MPS II progression more precociously than splenomegaly. In fact, in the pre-ERT phase of the analysis hepatomegaly was described in 58% of the patients of group A and splenomegaly in 9% of them; in group B 100% of the patients presented hepatomegaly and 33% splenomegaly; in group C 71% of the patients presented both hepato- and splenomegaly at start of treatment. Hepato/splenomegaly is widely associated to the disease and monitored as primary endpoint of ERT efficacy in MPS II patients [9]. In our analysis, a significant reduction of hepatomegaly was registered for patients of group B, while we did not detect a statistically significant reduction of splenomegaly in all 3 groups of patients after treatment. This may be partly due to the absence of spleen enlargement in most of the patients examined in the pre-ERT phase, which importantly reduced the size of analysable samples to 10 subjects. The recent study by the British HTA [15] did not detect, in a population of more than 20 patients, any significant reduction of both hepato- and splenomegaly, evaluated separately. However, such analysis was conducted on a follow-up of 12 and 24 months, while in our study final evaluations were performed on average 3 years after the start of ERT. In addition, the HTA evaluation on hepato/splenomegaly was conducted by palpation, which might be influenced by a certain degree of subjectivity; our examination was always confirmed by an ultrasound evaluation of the abdomen.

As for the heart involvement, clearly described for the disease in the vast majority of the patients [25], we primarily examined valvulopathies, which are the most frequently assessed cardiac disease in MPS patients [26]. In particular, we assessed valve regurgitation, a parameter more objectively evaluated than other cardiac measurements, and observed that even most of the youngest patients were already compromised in the pre-ERT analysis. Therefore, it would be useful to include valvulopathy among the signs to consider in the clinical suspect of Hunter Syndrome. According to our study, this sign, seems to slightly benefit from the treatment, leading to an improvement or to a stabilization of the valve regurgitation (in particular of the mitral and of the tricuspid valves), differently from what is reported by recent studies, in which ERT seems to significantly reduce the cardiac hypertrophy, but has limited effect on valve regurgitation [15,27]. However, we need to take into consideration that cardiac valves are connective tissue, thus missing an important blood and ERT supply. In addition, heart disease in these patients is often treated with other concomitant palliative medications; this could hamper the correct analysis and the detection of a potential ERT efficacy on this organ.

Respiratory evaluation in itself is sometimes difficult to carry on in these patients since FVC is applicable only to “attenuated”, collaborating subjects. Other parameters, strictly associated to the respiratory tract analysis, but not requiring or requiring less collaboration by the patients, were analysed in the present study, such as otological disorders including deafness, adenotonsillary hypertrophy and sleep disorders. None of them seemed to receive significant improvements by ERT administration.

Growth retardation is a peculiar feature of Hunter Syndrome. In MPS II patients, height is normal up to approximately 8–10 years of age and then it gradually decreases attesting on values below the third percentile [28-31]. Similarly, weight falls within the reference standards up to about 15 years of age, showing later on a negative trend [29,30]. In the present study growth patterns were analysed in terms of both height and weight. Height z-scores of Hunter patients under ERT, plotted vs treatment duration, showed that the youngest patients remained within normal values, ranging between −1 and +1 z-scores, but with the tendency to improve slightly over time. For group B the trend is negative with z-scores ranging from −3.5 to 1.5 with data distribution similar to that reported by Jones [31], for a group of patients aged 8–15 years, in the post-ERT period. The analysis of weight data confirms the negative trend described for this anthropometric variable in untreated Hunter patients [28-31] but suggests a potential reduction of this negative pattern mediated by ERT. This last observation could be particularly interesting since up today no data on the effect of idursulfase on weight are reported in the literature. Unfortunately, since control paired data on untreated patients were not available, a statistical analysis on the effect of ERT on both height and weight was not feasible.

Joint stiffness is another typical sign of Hunter Disease [32]. Evaluation performed in our population at baseline level showed a deep involvement of both upper and lower limbs in all patients. Improvements observed after treatment involved mainly the upper limbs, specifically abduction movements, with some stabilization, while no improvements were described for the lower limbs. Amelioration of upper limbs is of great importance for patients, who may acquire some autonomy in their daily functions, as well as for their families and caregivers. This improvement of potential performances may therefore determine an improvement of their quality of life.

The 6MWT test is considered a measure of functional capacity [9] and has been recently re-allocated from the secondary to the primary outcome evaluations for Hunter patients on ERT [33,34], although a significant limitation to its use is the poor compliance of Hunter patients [24]. Muscular-skeletal system is one of the districts involved in the 6MWT, together with heart, brain and respiratory functions. Unfortunately, in our study such evaluation could be performed only for a few patients; also, most of them was affected by a severe form of the disease. Our experience suggests that improvements of this test might be positively influenced mainly by respiratory function since both lower limbs extensibility and CNS may benefit from ERT to a lesser extent. Therefore, an amelioration of the respiratory functions may also be investigated by administering the 6MWT in compliant patients.

MPS II presents an important clinical heterogeneity; traditionally two phenotypes, attenuated and severe, have been reported. The distinguishing factor between the two forms is the presence, or absence, of progressive intellectual deterioration [4]. In a recent detailed description of a large cohort of patients, the down-slanting course was evident by 60 months of age at the latest, usually preceded by a plateau phase [35]. Approximately two thirds of the patients with diagnosis of MPS II will develop progressive neurodegeneration [36]. In a recent study of 36 Italian patients, some of the common brain and spinal cord features [revealed by Magnetic Resonance Imaging (MRI) and/or Computerized Tomography Scanning (CT-scan)] as WMAs, atrophy and/or communicating hydrocephalus and cranial vault hyperostosis, were observed to correlate with the severe Hunter phenotype. An enlarged cisterna magna was observed more frequently in the attenuated phenotype [5]. In the same study, ERT introduction did not seem to modify the disease progression in terms of WMAs, atrophy/communicating hydrocephalus and spinal stenosis. Our data confirm the inefficacy of ERT on the brain imaging parameters evaluated. Seizures are common neurologic complications in patients with MPS II. Schwartz and colleagues in a large cohort of 77 patients with Hunter Disease reported an incidence of 13% for seizures. They described a higher recurrence in subjects with the severe form, after the age of 10 years [28]. Seven of the patients of our study (26%) presented epilepsy, either pre-existent or with onset after the start of ERT. All of them were affected by the severe form of the disease and ERT did not show a positive effect on seizure. However, effects of ERT on CNS were not expected due to the inability of Elaprase^®^ to cross the Blood–brain Barrier (BBB) [37] and the recent multicentre study by Manara and colleagues [5] stated that even tissues which do not localize beyond the BBB, as head bones and meninges, do not seem to benefit from ERT.

To minimize biases caused by the small sample size of the 3 groups examined separately, we also evaluated all subjects enrolled, taken together as one group (n = 27), and all children up to 12 years of age at start of ERT (n = 20). However, these evaluations did not significantly change the results obtained on the clinical parameters examined with respect to the analysis conducted on the 3 separate groups.

Finally, ERT efficacy was also evaluated in relation to the severity of the disease, distinguishing “severe” (n = 17) from “attenuated” (n = 10) phenotypes. The analysis showed a slightly higher improvement/stabilization of parameters as GAG level, hepatomegaly, splenomegaly, otological disorders, adenotonsillar hypertrophy and cardiac valve regurgitation (except mitral valve) in severe vs attenuated patients. This may be due to the presence of more advanced clinical signs in severe patients at start of treatment, for which an amelioration due to ERT could result more evident. Again, no differences between the two groups were detected for joint stiffness and CNS abnormalities by brain imaging, confirming that ERT does not efficiently act on these clinical signs. In addition, our study confirmed that attenuated patients, together with no cognitive progressive deterioration, present no seizures. These findings should be more deeply evaluated to state if they represent sufficient evidence to exclude a prognosis of severe progression of the disease.

Strict monitoring of patients on ERT should be designed according to specific shared guidelines stating timing/frequency of specific evaluations, with the final aim to differentiate those subjects who may benefit from ERT from those who probably will not. Such monitoring is becoming mandatory for clinical, ethical and economic reasons. The high costs of orphan drugs and in particular of orphan biopharmaceuticals is not a new issue, as stated recently by some articles [38,39]. The average cost of idursulfase treatment in Italy exceeds 300.000 €/year for a 30 kg patient and proportionally increases with patients’ weight; compared to this, the costs of treatment monitoring appear almost insignificant and necessary to assess drug efficacy in every single patient. The identification of other biological markers, easy to access and affordable, to investigate the response to therapy when correlated to clear clinical signs, should be strongly encouraged.

Finally, as recently suggested for the severe form of the disease, possible criteria of discontinuation of the therapy should be identified and shared by caring physicians, and clearly explained to the family before ERT is initiated [40].

## Conclusions

In our Hunter population, Enzyme Replacement performed earlier than 12 years of age did not show to determine any more efficacy than when applied at an older age, except for urinary GAG, significantly reduced only in the younger groups, likely due to the low number of patients examined for the oldest group of age. No differences between the 2 populations were registered for hepatomegaly or splenomegaly, showing an identical proportion of positive outcomes. Some other parameters evaluated showed to take advantage from the treatment, although not significantly.

Also, efficacy of ERT in Hunter patients has revealed to be extremely subjective, despite a widely accepted common protocol (same dose/kg of body weight, time-schedule and velocity of infusion). Therefore, both dose and frequency regimens of administration likely need to be tailored to every single patient, based on his clinical picture.

## Additional files

Additional file 1
**Analysis of ENT manifestations.** Tables reporting the statistical analysis of otological disorders (group A: n = 9, group B: n = 3, group C: n = 6), adenotonsillar hypertrophy (group A: n = 6, group B: n = 1, group C: n = 2) and sleep disorders of respiratory origin (group A: n = 8, group B: n = 3, group C: n = 5). Additional file 2
**Analysis of weight z-scores.** Regression plot for weight z-scores before the start of ERT (time-point = 0) and after the start of ERT (time-points t >0) for patient groups aged 0–5 years (n = 11) (A) and aged 6–12 years (n = 7) (B). Additional file 3
**Analysis of neurological compartment.** Tables reporting the statistical analysis of neurological data on brain imaging (group A: n = 7, group B: n = 6, group C: n = 6), cognitive tests (group A: n = 11, group B: n = 6, group C: n = 3), and seizures (group A: n = 5, group B: n = 2, group C: n = 4). Additional file 4
**Analysis of severe versus attenuated patients.** Statistical analysis tables of clinical variables for severe and attenuated patients. Urinary GAG analysis: severe: n = 16, attenuated: n = 9; Hepatomegaly: severe: n = 15, attenuated: n = 10; Splenomegaly: severe: n = 13, attenuated: n = 9; Cardiac valves regurgitation: severe: n = 15 (n = 14 for mitral and aortic valves), attenuated: n = 7; Otological disorders: severe: n = 12, attenuated: n = 6; Adenotonsillar hypertrophy: severe: n = 10, attenuated: n = 6; Sleep disorders: severe: n = 10, attenuated: n = 6; Joint stiffness: severe: n = 15, attenuated: n = 7; CNS abnormalities by brain imaging: severe: n = 12, attenuated: n = 7; Cognitive function involvement: severe: n = 11, attenuated: n = 9; Seizure: severe: n = 9, attenuated: n = 2.

## Abbreviations

6MWT: 6-Minute Walk Test
BBB: Blood–brain Barrier
CNS: Central Nervous System
CRF: Case Report Form(s)
EC: Ethical Committee
ENT: Ear Nose Throat
ERT: Enzyme Replacement Therapy
FVC: Forced Vital Capacity
GAG: Glycosaminoglycans
HOS: Hunter Outcome Survey
IDS: Iduronate 2-sulfatase
MPS II: Mucopolysaccharidosis type II
MPS: Mucopolysaccharidosis
MRI: Magnetic Resonance Imaging
WMA: White Matter Abnormality(ies)

## References

[1] Beck M, Wijburg FA, Gal A (2012). Clinical utility gene card for: mucopolysaccharidosis type II. Eur J Hum Genet.

[2] Martin R, Beck M, Eng C, Giugliani R, Harmatz P, Munoz V, Muenzer J (2008). Recognition and diagnosis of mucopolysaccharidosis II (Hunter syndrome). Pediatrics.

[3] Neufeld EF, Muenzer J, Scriver CR, Beaudet AL, Sly WS (2001). The mucopolysaccharidoses. The Metabolic and Molecular Bases of Inherited Disease.

[4] Young ID, Harper PS, Newcombe RG, Archer IM (1982). A clinical and genetic study of Hunter’s syndrome. 2. Differences between the mild and severe forms. J Med Genet.

[5] Manara R, Priante E, Grimaldi M, Santoro L, Astarita L, Barone R, Concolino D, Di Rocco M, Donati MA, Fecarotta S, Ficcadenti A, Fiumara A, Furlan F, Giovannini I, Lilliu F, Mardari R, Polonara G, Procopio E, Rampazzo A, Rossi A, Sanna G, Parini R, Scarpa M (2011). Brain and spine MRI features of Hunter disease: frequency, natural evolution and response to therapy. J Inherit Metab Dis.

[6] Coppa GV, Gabrielli O, Zampini L, Jetzequel AM, Miniero R, Busca A, De Luca T, Di Natale P (1995). Bone marrow transplantation in Hunter syndrome. J Inherit Metab Dis.

[7] Guffon N, Bertrand Y, Forest I, Fouilhoux A, Froissart R (2009). Bone marrow transplantation in children with Hunter syndrome: outcome after 7 to 17 years. J Pediatr.

[8] Muenzer J (2014). Early initiation of enzyme replacement therapy for the mucopolysaccharidoses. Mol Genet Metab.

[9] Muenzer J, Wraith JE, Beck M, Giugliani R, Harmatz P, Eng CM, Vellodi A, Martin R, Ramaswami U, Gucsavas-Calikoglu M, Vijayaraghavan S, Wendt S, Puga AC, Ulbrich B, Shinawi M, Cleary M, Piper D, Conway AM, Kimura A (2006). A phase II/III clinical study of enzyme replacement therapy with idursulfase in mucopolysaccharidosis II (Hunter syndrome). Genet Med.

[10] Muenzer J, Gucsavas-Calikoglu M, McCandless SE, Schuetz TJ, Kimura A (2007). A phase I/II clinical trial of enzyme replacement therapy in mucopolysaccharidosis II (Hunter syndrome). Mol Genet Metab.

[11] Alcalde-Martin C, Muro-Tudelilla JM, Cancho-Candela R, Gutierrez-Solana LG, Pintos-Morell G, Marti-Herrero M, Munguira-Aguado P, Galan-Gomez E (2010). First experience of enzyme replacement therapy with idursulfase in Spanish patients with Hunter syndrome under 5 years of age: case observations from the Hunter Outcome Survey (HOS). Eur J Med Genet.

[12] Muenzer J, Beck M, Giugliani R, Suzuki Y, Tylki-Szymanska A, Valayannopoulos V, Vellodi A, Wraith JE (2011). Idursulfase treatment of Hunter syndrome in children younger than 6 years: results from the Hunter Outcome Survey. Genet Med.

[13] Giugliani R, Hwu WL, Tylki-Szymanska A, Whiteman DA, Pano A (2014). A multicenter, open-label study evaluating safety and clinical outcomes in children (1.4-7.5 years) with Hunter syndrome receiving idursulfase enzyme replacement therapy. Genet Med.

[14] Lampe C, Atherton A, Burton BK, Descartes M, Giugliani R, Horovitz DD, Kyosen SO, Magalhaes TS, Martins AM, Mendelsohn NJ, Muenzer J, Smith LD: **Enzyme Replacement Therapy in Mucopolysaccharidosis II Patients Under 1 Year of Age.** *JIMD Rep* 2014, Epub ahead of print. 10.1007/8904_2013_289 PMC4213327 24515576

[15] Wyatt K, Henley W, Anderson L, Anderson R, Nikolaou V, Stein K, Klinger L, Hughes D, Waldek S, Lachmann R, Mehta A, Vellodi A, Logan S (2012). The effectiveness and cost-effectiveness of enzyme and substrate replacement therapies: a longitudinal cohort study of people with lysosomal storage disorders. Health Technol Assess.

[16] Vellodi A, Wraith JE, Chakrapani A, Hendriksz C, Jones S, Lavery C: **Mucopolysaccharidosis type II: Guidelines for Assessment, Monitoring and Enzyme Replacement Therapy (ERT) - MPS II guidelines Version II – reviewed 2010.** [http://www.webarchive.org.uk/wayback/archive/20130328002531/http://www.specialisedservices.nhs.uk/library/23/Guidelines_for_Mucopolysaccharidosis_Type_II.pdf].

[17] Clopper CJ, Pearson ES (1934). The use of confidence or fiducial limits illustrated in the case of the binomial. Biometrika.

[18] Zoghbi WA, Enriquez-Sarano M, Foster E, Grayburn PA, Kraft CD, Levine RA, Nihoyannopoulos P, Otto CM, Quinones MA, Rakowski H, Stewart WJ, Waggoner A, Weissman NJ, American Society of Echocardiography (2003). Recommendations for evaluation of the severity of native valvular regurgitation with two-dimensional and Doppler echocardiography. J Am Soc Echocardiogr.

[19] Cole TJ, Green PJ (1992). Smoothing reference centile curves: the LMS method and penalized likelihood. Stat Med.

[20] Lammers AE, Hislop AA, Flynn Y, Haworth SG (2008). The 6-minute walk test: normal values for children of 4–11 years of age. Arch Dis Child.

[21] Muenzer J, Beck M, Eng CM, Giugliani R, Harmatz P, Martin R, Ramaswami U, Vellodi A, Wraith JE, Cleary M, Gucsavas-Calikoglu M, Puga AC, Shinawi M, Ulbrich B, Vijayaraghavan S, Wendt S, Conway AM, Rossi A, Whiteman DA, Kimura A (2011). Long-term, open-labeled extension study of idursulfase in the treatment of Hunter syndrome. Genet Med.

[22] Scarpa M, Pagon RA, Bird TD, Dolan CR, Stephens K, Adam MP (1993). Mucopolysaccharidosis Type II. GeneReviews.

[23] Scarpa M, Almassy Z, Beck M, Bodamer O, Bruce IA, De Meirleir L, Guffon N, Guillen-Navarro E, Hensman P, Jones S, Kamin W, Kampmann C, Lampe C, Lavery CA, Teles EL, Link B, Lund AM, Malm G, Pitz S, Rothera M, Stewart C, Tylki-Szymanska A, Van der Ploeg A, Walker R, Zeman J, Wraith JE, Hunter Syndrome Europena Expert Council (2011). Mucopolysaccharidosis type II: European recommendations for the diagnosis and multidisciplinary management of a rare disease. Orphanet J Rare Dis.

[24] Glamuzina E, Fettes E, Bainbridge K, Crook V, Finnegan N, Abulhoul L, Vellodi A (2011). Treatment of mucopolysaccharidosis type II (Hunter syndrome) with idursulfase: the relevance of clinical trial end points. J Inherit Metab Dis.

[25] Kampmann C, Beck M, Morin I, Loehr JP (2011). Prevalence and characterization of cardiac involvement in Hunter syndrome. J Pediatr.

[26] Fesslova V, Corti P, Sersale G, Rovelli A, Russo P, Mannarino S, Butera G, Parini R (2009). The natural course and the impact of therapies of cardiac involvement in the mucopolysaccharidoses. Cardiol Young.

[27] Brands MM, Frohn-Mulder IM, Hagemans ML, Hop WC, Oussoren E, Helbing WA, van der Ploeg AT (2013). Mucopolysaccharidosis: cardiologic features and effects of enzyme-replacement therapy in 24 children with MPS I, II and VI. J Inherit Metab Dis.

[28] Schwartz IV, Ribeiro MG, Mota JG, Toralles MB, Correia P, Horovitz D, Santos ES, Monlleo IL, Fett-Conte AC, Sobrinho RP, Norato DY, Paula AC, Kim CA, Duarte AR, Boy R, Valadares E, De Michelena M, Mabe P, Martinhago CD, Pina-Neto JM, Kok F, Leistner-Segal S, Burin MG, Giugliani R (2007). A clinical study of 77 patients with mucopolysaccharidosis type II. Acta Paediatr Suppl.

[29] Rozdzynska A, Tylki-Szymanska A, Jurecka A, Cieslik J (2011). Growth pattern and growth prediction of body height in children with mucopolysaccharidosis type II. Acta Paediatr.

[30] Wraith JE, Beck M, Giugliani R, Clarke J, Martin R, Muenzer J, HOS Investigators (2008). Initial report from the Hunter Outcome Survey. Genet Med.

[31] Jones SA, Parini R, Harmatz P, Giugliani R, Fang J, Mendelsohn NJ, HOS Natural History Working Group on behalf of HOS Investigators (2013). The effect of idursulfase on growth in patients with Hunter syndrome: data from the Hunter Outcome Survey (HOS). Mol Genet Metab.

[32] Marucha J, Jurecka A, Syczewska M, Rozdzynska-Swiatkowska A, Tylki-Szymanska A (2012). Restricted joint range of motion in patients with MPS II: correlation with height, age and functional status. Acta Paediatr.

[33] Da Silva EM, Strufaldi MW, Andriolo RB, Silva LA (2011). Enzyme replacement therapy with idursulfase for mucopolysaccharidosis type II (Hunter syndrome). Cochrane Database Syst Rev.

[34] Da Silva EM, Strufaldi MW, Andriolo RB, Silva LA (2014). Enzyme replacement therapy with idursulfase for mucopolysaccharidosis type II (Hunter syndrome). Cochrane Database Syst Rev.

[35] Holt JB, Poe MD, Escolar ML (2011). Natural progression of neurological disease in mucopolysaccharidosis type II. Pediatrics.

[36] Muenzer J, Beck M, Eng CM, Escolar ML, Giugliani R, Guffon NH, Harmatz P, Kamin W, Kampmann C, Koseoglu ST, Link B, Martin RA, Molter DW, Munoz Rojas MV, Ogilvie JW, Parini R, Ramaswami U, Scarpa M, Schwartz IV, Wood RE, Wraith E (2009). Multidisciplinary management of Hunter syndrome. Pediatrics.

[37] Wraith JE, Scarpa M, Beck M, Bodamer OA, De Meirleir L, Guffon N, Meldgaard Lund A, Malm G, Van der Ploeg AT, Zeman J (2008). Mucopolysaccharidosis type II (Hunter syndrome): a clinical review and recommendations for treatment in the era of enzyme replacement therapy. Eur J Pediatr.

[38] Roos JC, Hyry HI, Cox TM (2010). Orphan drug pricing may warrant a competition law investigation. BMJ.

[39] Simoens S (2011). Pricing and reimbursement of orphan drugs: the need for more transparency. Orphanet J Rare Dis.

[40] Muenzer J, Bodamer O, Burton B, Clarke L, Frenking GS, Giugliani R, Jones S, Rojas MV, Scarpa M, Beck M, Harmatz P (2012). The role of enzyme replacement therapy in severe Hunter syndrome-an expert panel consensus. Eur J Pediatr.

