# Supplementary material for: Clinical efficacy of Enzyme Replacement Therapy in paediatric Hunter patients, an independent study of 3.5 years
Source: Orphanet J Rare Dis. 2014 Sep 18;9:129. doi: 10.1186/s13023-014-0129-1 (PMC4180060; doi:10.1186/s13023-014-0129-1)
Supplement: Additional file 1 — Analysis of ENT manifestations. Tables reporting the statistical analysis of otological disorders (group A: n = 9, group B: n = 3, group C: n = 6), adenotonsillar hypertrophy (group A: n = 6, group B: n = 1, group C: n = 2) and sleep disorders of respiratory origin (group A: n = 8, group B: n = 3, group C: n = 5). [file 13023_2014_129_MOESM1_ESM.docx]

**Additional File 1**

**Otological disorders**

|  |  |  |  |  | **POST** | | **McNemar test**  **p-value** |  | **Positive Outcomes** | |
| --- | --- | --- | --- | --- | --- | --- | --- | --- | --- | --- |
|  |  |  |  |  | **Y** | **N** |  |  | **Proportion** | **CI (95%)** |
| **AGE GROUP** | **A** |  | **PRE** | **Y** | 4 | 2 | 1.0 |  | 0.44 | (0.137, 0.788) |
|  |  |  |  | **N** | 1 | 2 |  |  |  |  |
|  | **B** |  | **PRE** | **Y** | 2 | 0 | 1.0 |  | 0.33 | (0.008, 0.906) |
|  |  |  |  | **N** | 0 | 1 |  |  |  |  |
|  | **C** |  | **PRE** | **Y** | 1 | 2 | 1.0 |  | 0.67 | (0.223, 0.957) |
|  |  |  |  | **N** | 1 | 2 |  |  |  |  |
|  | **A+B** |  | **PRE** | **Y** | 6 | 2 | 1.0 |  | 0.42 | (0.152, 0.723) |
|  |  |  |  | **N** | 1 | 3 |  |  |  |  |
|  | **A+B+C** |  | **PRE** | **Y** | 7 | 4 | 0.69 |  | 0.50 | (0.261, 0.74) |
|  |  |  |  | **N** | 2 | 5 |  |  |  |  |

**Adenotonsillar hypertrophy**

|  |  |  |  |  | **POST** | | **McNemar test**  **p-value** |  | **Positive Outcomes** | |
| --- | --- | --- | --- | --- | --- | --- | --- | --- | --- | --- |
|  |  |  |  |  | **Y** | **N** |  |  | **Proportion** | **CI (95%)** |
| **AGE GROUP** | **A** |  | **PRE** | **Y** | 2 | 1 | 1.0 |  | 0.50 | (0.118, 0.882) |
|  |  |  |  | **N** | 1 | 2 |  |  |  |  |
|  | **B** |  | **PRE** | **Y** | 1 | 0 | 1.0 |  | 0.00 | (0, 0.975) |
|  |  |  |  | **N** | 0 | 0 |  |  |  |  |
|  | **C** |  | **PRE** | **Y** | 1 | 1 | 1.0 |  | 0.50 | (0.013, 0.987) |
|  |  |  |  | **N** | 0 | 0 |  |  |  |  |
|  | **A+B** |  | **PRE** | **Y** | 3 | 1 | 1.0 |  | 0.43 | (0.099, 0.816) |
|  |  |  |  | **N** | 1 | 2 |  |  |  |  |
|  | **A+B+C** |  | **PRE** | **Y** | 4 | 2 | 1.0 |  | 0.44 | (0.137, 0.788) |
|  |  |  |  | **N** | 1 | 2 |  |  |  |  |

**Sleep disorders of respiratory origin**

|  |  |  |  |  | **POST** | | **McNemar test**  **p-value** |  | **Positive Outcomes** | |
| --- | --- | --- | --- | --- | --- | --- | --- | --- | --- | --- |
|  |  |  |  |  | **Y** | **N** |  |  | **Proportion** | **CI (95%)** |
| **AGE GROUP** | **A** |  | **PRE** | **Y** | 2 | 0 | 1.0 |  | 0.63 | (0.245, 0.915) |
|  |  |  |  | **N** | 1 | 5 |  |  |  |  |
|  | **B** |  | **PRE** | **Y** | 2 | 0 | 1.0 |  | 0.33 | (0.008, 0.906) |
|  |  |  |  | **N** | 0 | 1 |  |  |  |  |
|  | **C** |  | **PRE** | **Y** | 2 | 0 | 1.0 |  | 0.60 | (0.147, 0.947) |
|  |  |  |  | **N** | 0 | 3 |  |  |  |  |
|  | **A+B** |  | **PRE** | **Y** | 4 | 0 | 1.0 |  | 0.55 | (0.234, 0.833) |
|  |  |  |  | **N** | 1 | 6 |  |  |  |  |
|  | **A+B+C** |  | **PRE** | **Y** | 6 | 0 | 1.0 |  | 0.56 | (0.299, 0.803) |
|  |  |  |  | **N** | 1 | 9 |  |  |  |  |
