# Supplementary material for: Clinical efficacy of Enzyme Replacement Therapy in paediatric Hunter patients, an independent study of 3.5 years
Source: Orphanet J Rare Dis. 2014 Sep 18;9:129. doi: 10.1186/s13023-014-0129-1 (PMC4180060; doi:10.1186/s13023-014-0129-1)
Supplement: Additional file 3 — Analysis of neurological compartment. Tables reporting the statistical analysis of neurological data on brain imaging (group A: n = 7, group B: n = 6, group C: n = 6), cognitive tests (group A: n = 11, group B: n = 6, group C: n = 3), and seizures (group A: n = 5, group B: n = 2, group C: n = 4). [file 13023_2014_129_MOESM3_ESM.docx]

**Additional File 3**

**Brain imaging**

|  |  |  |  |  | **POST** | | **McNemar test**  **p-value** |  | **Positive Outcomes** | |
| --- | --- | --- | --- | --- | --- | --- | --- | --- | --- | --- |
|  |  |  |  |  | **Y** | **N** |  |  | **Proportion** | **CI (95%)** |
| **AGE GROUP** | **A** |  | **PRE** | **Y** | 7 | 0 | 1.0 |  | 0.0 | (0, 0.41) |
|  |  |  |  | **N** | 0 | 0 |  |  |  |  |
|  | **B** |  | **PRE** | **Y** | 6 | 0 | 1.0 |  | 0.0 | (0, 0.459) |
|  |  |  |  | **N** | 0 | 0 |  |  |  |  |
|  | **C** |  | **PRE** | **Y** | 6 | 0 | 1.0 |  | 0.0 | (0, 0.459) |
|  |  |  |  | **N** | 0 | 0 |  |  |  |  |
|  | **A+B** |  | **PRE** | **Y** | 13 | 0 | 1.0 |  | 0.0 | (0, 0.247) |
|  |  |  |  | **N** | 0 | 0 |  |  |  |  |
|  | **A+B+C** |  | **PRE** | **Y** | 19 | 0 | 1.0 |  | 0.0 | (0, 0.177) |
|  |  |  |  | **N** | 0 | 0 |  |  |  |  |

**Cognitive impairment**

|  |  |  |  |  | **POST** | | **McNemar test**  **p-value** |  | **Positive Outcomes** | |
| --- | --- | --- | --- | --- | --- | --- | --- | --- | --- | --- |
|  |  |  |  |  | **Y** | **N** |  |  | **Proportion** | **CI (95%)** |
| **AGE GROUP** | **A** |  | **PRE** | **Y** | 7 | 0 | 1.0 |  | 0.364 | (0.109, 0.692) |
|  |  |  |  | **N** | 0 | 4 |  |  |  |  |
|  | **B** |  | **PRE** | **Y** | 4 | 0 | 1.0 |  | 0.333 | (0.043, 0.777) |
|  |  |  |  | **N** | 0 | 2 |  |  |  |  |
|  | **C** |  | **PRE** | **Y** | 0 | 0 | 1.0 |  | 1.0 | (0.292, 1) |
|  |  |  |  | **N** | 0 | 3 |  |  |  |  |
|  | **A+B** |  | **PRE** | **Y** | 11 | 0 | 1.0 |  | 0.353 | (0.142, 0.617) |
|  |  |  |  | **N** | 0 | 9 |  |  |  |  |
|  | **A+B+C** |  | **PRE** | **Y** | 11 | 0 | 1.0 |  | 0.45 | (0.231, 0.685) |
|  |  |  |  | **N** | 0 | 6 |  |  |  |  |

**Seizure**

|  |  |  |  |  | **POST** | | **McNemar test**  **p-value** |  | **Positive Outcomes** | |
| --- | --- | --- | --- | --- | --- | --- | --- | --- | --- | --- |
|  |  |  |  |  | **Y** | **N** |  |  | **Proportion** | **CI (95%)** |
| **AGE GROUP** | **A** |  | **PRE** | **Y** | 0 | 0 | 0.5 |  | 0.6 | (0.147, 0.947) |
|  |  |  |  | **N** | 2 | 3 |  |  |  |  |
|  | **B** |  | **PRE** | **Y** | 2 | 0 | 1.0 |  | 0.0 | (0, 0.842) |
|  |  |  |  | **N** | 0 | 0 |  |  |  |  |
|  | **C** |  | **PRE** | **Y** | 2 | 0 | 1.0 |  | 0.5 | (0.068, 0.932) |
|  |  |  |  | **N** | 0 | 2 |  |  |  |  |
|  | **A+B** |  | **PRE** | **Y** | 4 | 0 | 0.5 |  | 0.429 | (0.099, 0.816) |
|  |  |  |  | **N** | 2 | 5 |  |  |  |  |
|  | **A+B+C** |  | **PRE** | **Y** | 2 | 0 | 0.5 |  | 0.455 | (0.168, 0.766) |
|  |  |  |  | **N** | 2 | 3 |  |  |  |  |
