# Supplementary material for: Clinical efficacy of Enzyme Replacement Therapy in paediatric Hunter patients, an independent study of 3.5 years
Source: Orphanet J Rare Dis. 2014 Sep 18;9:129. doi: 10.1186/s13023-014-0129-1 (PMC4180060; doi:10.1186/s13023-014-0129-1)
Supplement: Additional file 4 — Analysis of severe versus attenuated patients. Statistical analysis tables of clinical variables for severe and attenuated patients. Urinary GAG analysis: severe: n = 16, attenuated: n = 9; Hepatomegaly: severe: n = 15, attenuated: n = 10; Splenomegaly: severe: n = 13, attenuated: n = 9; Cardiac valves regurgitation: severe: n = 15 (n = 14 for mitral and aortic valves), attenuated: n = 7; Otological disorders: severe: n = 12, attenuated: n = 6; Adenotonsillar hypertrophy: severe: n = 10, attenuated: n = 6; Sleep disorders: severe: n = 10, attenuated: n = 6; Joint stiffness: severe: n = 15, attenuated: n = 7; CNS abnormalities by brain imaging: severe: n = 12, attenuated: n = 7; Cognitive function involvement: severe: n = 11, attenuated: n = 9; Seizure: severe: n = 9, attenuated: n = 2. [file 13023_2014_129_MOESM4_ESM.docx]

**Additional File 4**

**Urinary GAG analysis**

| **Severity** | **Time point from start of ERT** | **n** | **Median**  **(minimum-maximum)** | **p-value** |
| --- | --- | --- | --- | --- |
| **Severe** | 1 yr ± 4 m | 16 | 31.3 (7.8-88.1) | <.0001 |
|  | 2 yrs ± 4 m | 13 | 18.7 (3.9-92.1) | 0.0002 |
|  | 3 yrs ± 4 m | 10 | 20.6 (5.7-81.8) | 0.0020 |
|  | Last | 16 | 16.4 (5.1-79.2) | <.0001 |
| **Attenuated** | 1 yr ± 4 m | 7 | 38.8 (25.9-50.4) | 0.0156 |
|  | 2 yrs ± 4 m | 4 | 24.7 (18.6-30.1) | 0.1250 |
|  | 3 yrs ± 4 m | 6 | 26.3 (13.7-54.2) | 0.0313 |
|  | Last | 9 | 29.4 (14.4-56.9) | 0.0039 |

**Hepatomegaly**

|  |  |  |  |  | **POST** | | **McNemar test**  **p-value** |  | **Positive Outcomes** | |
| --- | --- | --- | --- | --- | --- | --- | --- | --- | --- | --- |
|  |  |  |  |  | **Y** | **N** |  |  | **Proportion** | **CI (95%)** |
| **SEVERITY GROUP** | **Severe** |  | **PRE** | **Y** | 4 | 8 | 0.109 |  | 0.600 | (0.323, 0.837) |
|  |  |  |  | **N** | 2 | 1 |  |  |  |  |
|  | **Attenuated** |  | **PRE** | **Y** | 3 | 3 | 1.0 |  | 0.500 | (0.187, 0.813) |
|  |  |  |  | **N** | 2 | 2 |  |  |  |  |

**Splenomegaly**

|  |  |  |  |  | **POST** | | **McNemar test**  **p-value** |  | **Positive Outcomes** | |
| --- | --- | --- | --- | --- | --- | --- | --- | --- | --- | --- |
|  |  |  |  |  | **Y** | **N** |  |  | **Proportion** | **CI (95%)** |
| **SEVERITY GROUP** | **Severe** |  | **PRE** | **Y** | 2 | 5 | 0.219 |  | 0.769 | (0.462, 0.95) |
|  |  |  |  | **N** | 1 | 5 |  |  |  |  |
|  | **Attenuated** |  | **PRE** | **Y** | 2 | 1 | 1.0 |  | 0.556 | (0.212, 0.863) |
|  |  |  |  | **N** | 2 | 4 |  |  |  |  |

**Cardiac valves regurgitation**

**Otological disorders**

|  |  |  |  |  | **POST** | | **McNemar test**  **p-value** |  | **Positive Outcomes** | |
| --- | --- | --- | --- | --- | --- | --- | --- | --- | --- | --- |
|  |  |  |  |  | **Y** | **N** |  |  | **Proportion** | **CI (95%)** |
| **SEVERITY GROUP** | **Severe** |  | **PRE** | **Y** | 4 | 4 | 0.375 |  | 0.583 | (0.277, 0.848) |
|  |  |  |  | **N** | 1 | 3 |  |  |  |  |
|  | **Attenuated** |  | **PRE** | **Y** | 3 | 0 | 1.0 |  | 0.333 | (0.043, 0.777) |
|  |  |  |  | **N** | 1 | 2 |  |  |  |  |

**Adenotonsillar hypertrophy**

|  |  |  |  |  | **POST** | | **McNemar test**  **p-value** |  | **Positive Outcomes** | |
| --- | --- | --- | --- | --- | --- | --- | --- | --- | --- | --- |
|  |  |  |  |  | **Y** | **N** |  |  | **Proportion** | **CI (95%)** |
| **SEVERITY GROUP** | **Severe** |  | **PRE** | **Y** | 1 | 2 | 0.5 |  | 0.75 | (0.194, 0.994) |
|  |  |  |  | **N** | 0 | 1 |  |  |  |  |
|  | **Attenuated** |  | **PRE** | **Y** | 3 | 0 | 1.0 |  | 0.2 | (0.005, 0.716) |
|  |  |  |  | **N** | 1 | 1 |  |  |  |  |

**Sleep disorders**

|  |  |  |  |  | **POST** | | **McNemar test**  **p-value** |  | **Positive Outcomes** | |
| --- | --- | --- | --- | --- | --- | --- | --- | --- | --- | --- |
|  |  |  |  |  | **Y** | **N** |  |  | **Proportion** | **CI (95%)** |
| **SEVERITY GROUP** | **Severe** |  | **PRE** | **Y** | 6 | 0 | 1.0 |  | 0.3 | (0.067, 0.653) |
|  |  |  |  | **N** | 1 | 3 |  |  |  |  |
|  | **Attenuated** |  | **PRE** | **Y** | 0 | 0 | 1.0 |  | 1.0 | (0.541, 1) |
|  |  |  |  | **N** | 0 | 6 |  |  |  |  |

**Joint stiffness**

|  |  |  |  |  | **POST** | | **McNemar test**  **p-value** |  | **Positive Outcomes** | |
| --- | --- | --- | --- | --- | --- | --- | --- | --- | --- | --- |
|  |  |  |  |  | **Y** | **N** |  |  | **Proportion** | **CI (95%)** |
| **SEVERITY GROUP** | **Severe** |  | **PRE** | **Y** | 13 | 1 | 1.0 |  | 0.067 | (0.002, 0.32) |
|  |  |  |  | **N** | 1 | 0 |  |  |  |  |
|  | **Attenuated** |  | **PRE** | **Y** | 7 | 0 | 1.0 |  | 0.0 | (0, 0.41) |
|  |  |  |  | **N** | 0 | 0 |  |  |  |  |

**CNS abnormalities by brain imaging**

|  |  |  |  |  | **POST** | | **McNemar test**  **p-value** |  | **Positive Outcomes** | |
| --- | --- | --- | --- | --- | --- | --- | --- | --- | --- | --- |
|  |  |  |  |  | **Y** | **N** |  |  | **Proportion** | **CI (95%)** |
| **SEVERITY GROUP** | **Severe** |  | **PRE** | **Y** | 12 | 0 | 1.0 |  | 0.0 | (0, 0.265) |
|  |  |  |  | **N** | 0 | 0 |  |  |  |  |
|  | **Attenuated** |  | **PRE** | **Y** | 7 | 0 | 1.0 |  | 0.0 | (0, 0.41) |
|  |  |  |  | **N** | 0 | 0 |  |  |  |  |

**Cognitive function involvement**

|  |  |  |  |  | **POST** | | **McNemar test**  **p-value** |  | **Positive Outcomes** | |
| --- | --- | --- | --- | --- | --- | --- | --- | --- | --- | --- |
|  |  |  |  |  | **Y** | **N** |  |  | **Proportion** | **CI (95%)** |
| **SEVERITY GROUP** | **Severe** |  | **PRE** | **Y** | 11 | 0 | 1.0 |  | 0.0 | (0, 0.285) |
|  |  |  |  | **N** | 0 | 0 |  |  |  |  |
|  | **Attenuated** |  | **PRE** | **Y** | 0 | 0 | 1.0 |  | 1.0 | (0.664, 1) |
|  |  |  |  | **N** | 0 | 9 |  |  |  |  |

**Seizure**

|  |  |  |  |  | **POST** | | **McNemar test**  **p-value** |  | **Positive Outcomes** | |
| --- | --- | --- | --- | --- | --- | --- | --- | --- | --- | --- |
|  |  |  |  |  | **Y** | **N** |  |  | **Proportion** | **CI (95%)** |
| **SEVERITY GROUP** | **Severe** |  | **PRE** | **Y** | 4 | 0 | 0.5 |  | 0.333 | (0.075, 0.701) |
|  |  |  |  | **N** | 2 | 3 |  |  |  |  |
|  | **Attenuated** |  | **PRE** | **Y** | 0 | 0 | 1.0 |  | 1.0 | (0.158, 1) |
|  |  |  |  | **N** | 0 | 2 |  |  |  |  |
